# Roles of Mitochondrial Dysfunction in Diabetic Kidney Disease: New Perspectives from Mechanism to Therapy

**DOI:** 10.3390/biom14060733

**Published:** 2024-06-20

**Authors:** Yichen Yang, Jiahui Liu, Qiling Shi, Buyu Guo, Hanbing Jia, Yuxuan Yang, Songbo Fu

**Affiliations:** 1 The First Clinical Medical College, Lanzhou University, Lanzhou 730000, China; ychyang2021@lzu.edu.cn (Y.Y.); jhliu21@lzu.edu.cn (J.L.); guoby21@lzu.edu.cn (B.G.); 320220910600@lzu.edu.cn (H.J.); yxyang2020@lzu.edu.cn (Y.Y.); 2 Department of Endocrinology, First Hospital of Lanzhou University, Lanzhou 730000, China; 3 The Second Clinical Medical College, Lanzhou University, Lanzhou 730000, China; shiql20@lzu.edu.cn; 4 Gansu Provincial Endocrine Disease Clinical Medicine Research Center, Lanzhou 730000, China

**Keywords:** diabetic nephropathy, mitochondrial dysfunction, microangiopathy, oxidative stress, treatment

## Abstract

Diabetic kidney disease (DKD) is a common microvascular complication of diabetes and the main cause of end-stage renal disease around the world. Mitochondria are the main organelles responsible for producing energy in cells and are closely involved in maintaining normal organ function. Studies have found that a high-sugar environment can damage glomeruli and tubules and trigger mitochondrial dysfunction. Meanwhile, animal experiments have shown that DKD symptoms are alleviated when mitochondrial damage is targeted, suggesting that mitochondrial dysfunction is inextricably linked to the development of DKD. This article describes the mechanisms of mitochondrial dysfunction and the progression and onset of DKD. The relationship between DKD and mitochondrial dysfunction is discussed. At the same time, the progress of DKD treatment targeting mitochondrial dysfunction is summarized. We hope to provide new insights into the progress and treatment of DKD.

## 1. Introduction

Diabetic kidney disease (DKD) is one of the most serious complications of diabetic microangiopathy and is considered to be the leading cause of death in people with diabetes. The report “Diabetes and Kidney Disease”, published by the International Diabetes Federation in 2023, states that chronic kidney disease caused by type 2 diabetes increased by up to 74% globally from 1990 to 2017. Meanwhile, the prevalence of DKD in diabetic patients varies by country and region, with the United States and the Middle East being the most severely affected regions [1]. DKD is caused by chronic hyperglycemia and is characterized by albuminuria, thickening of the glomerular and tubular basement membranes, and glomerulosclerosis [2]. DKD can significantly delay the progression of diabetes through early intervention, early diagnosis, and intensive diabetes management. The current clinical treatment methods mainly include targeting the renin–angiotensin system and using aldosterone receptor antagonists [3]. Recent exploration and advancements in the molecular and cellular fields have also provided additional therapeutic targets for DKD [2].

As one of the organs with a high energy demand in the human body, the kidney needs a large number of mitochondria to provide enough energy to maintain normal physiological functions. Mitochondria are organelles surrounded by two lipid bilayers that provide energy to the kidneys by producing ATP. The normal operation of this function is closely related to the homeostasis of mitochondria, antioxidant capacity, and nutritional pathways in the kidney. Therefore, damage to any link may lead to mitochondrial dysfunction [4]. In recent years, the types of diseases defined by mitochondrial dysfunction have gradually increased in number and include metabolic diseases, cardiovascular diseases, and neurodegenerative diseases [5]. A large number of experimental studies suggest that mitochondrial dysfunction is one of the important causes of DKD, and restoring mitochondrial function may effectively alleviate kidney damage caused by DKD [6]. This suggests that mitochondrial dysfunction may be crucial in the progression of DKD.

In this review, we provide a brief overview of the mechanisms of mitochondrial dysfunction as well as the pathogenesis of DKD. We then explore the relationship between mitochondrial dysfunction and glomerular and tubular lesions in the course of DKD. We also focus on reviewing the research progress in targeting mitochondrial dysfunction for the treatment of DKD, with the expectation of providing new insights for the clinical treatment of DKD.

## 2. Mechanisms of Mitochondrial Dysfunction

The main physiological function of mitochondria is to produce ATP through oxidative phosphorylation, while other functions include mitochondrial biogenesis, reactive oxygen species production, autophagy, fusion, and lysis. Damage to mitochondria caused by any of these aspects can be referred to as mitochondrial dysfunction. In the following, we will discuss the main mechanisms of mitochondrial dysfunction, mainly from the above four aspects.

Mitochondrial biogenesis is the physiological response to external stimuli caused by interactions between the mitochondrial and nuclear genomes. It is also involved in the regulation of mitochondrial replication, transcription, translation, and other aspects of protein and DNA synthesis intended to maintain mitochondrial homeostasis [7]. Currently, it is believed that mitochondrial biogenesis is closely related to peroxisome proliferator-activated receptor-γ coactivator α (PGC-1α), nuclear respiratory factors (NRF-1 and NRF-2), mitochondrial transcription factor A (mtTFA), etc. [8]. PGC-1α is a central factor in biogenesis. It regulates the pathway of mitochondrial function through regulators such as AMP-activated protein kinase and cAMP response element-binding protein (CREB). PGC1-α can also further activate NRF1 and TFAM downstream to promote mitochondrial generation [9]. Moreover, PGC-1α is also regulated by post-translational modifications such as phosphorylation, methylation, acetylation, and ubiquitination [10]. It is precisely due to the complexity and importance of mitochondrial biogenesis that it has such a profound impact on the state of cellular metabolism.

Reactive oxygen species (ROS) are small-molecule compounds derived from oxygen, mainly produced by the mitochondrial electron respiratory chain. When the body uses a large amount of energy, this process is accompanied by the production of ROS during normal oxygen metabolism. The mitochondrial electron transport chain (ETC) is composed of complexes I–IV and the electron transport proteins ubiquinone and cytochrome c. Currently, it is believed that the formation of ATP can be achieved through two pathways, as part of which the electrons leaked from ETC interact with oxygen to produce ROS. Among the factors that affect ROS production, the NADPH oxidase family can transfer electrons across the plasma membrane and produce ROS through its homologs [11]. In the respiratory chain, ROS are readily generated during the transfer of electrons from NADH to ubiquinone in complex I and the acceptance of electrons from succinate by FAD in complexe II [12]. ROS are also regulated differently in different situations. PGC-1α, as a necessary substance for antioxidant enzymes under inflammatory conditions, can downregulate the production of ROS to alleviate inflammatory damage. The recognized stress transcription factor nuclear factor erythroid 2-related factor 2 (Nrf2) can reduce ROS production through antioxidant stress. In some immune defenses, ROS can be induced to increase in number by the overexpression of TNF, but excessive ROS can drive NF-ĸB downstream signal transduction, which is closely related to many complications of diabetes. Its importance in disease development is also determined by its characteristics as a signaling molecule and its exposure to multiple factors [13].

Autophagy is a major pathway for the degradation of proteins and intracellular organelles, and the process is largely dependent on lysosomes. Autophagy is thought to begin with the formation of a barrier membrane that may come from the rough endoplasmic reticulum. Subsequently, the isolation membrane envelops and engulfs the targeted substances inside the cell and fuses with lysosomes to form autophagic lysosomes, causing the intracellular substances to be degraded and recovered by hydrolytic enzymes [14]. Mitochondrial autophagy is a kind of selective autophagy that maintains the biological activity of mitochondria by selectively degrading damaged and aging mitochondria. Two key steps in the coordination mechanism of autophagy are ATG5–ATG12 coupling and LC3 processing, both of which are involved in the formation of autophagosomes under the action of ubiquitin-like systems. At the same time, LC3 synthesis is increased during autophagy, so it is commonly used to evaluate autophagy function [15]. Other molecules closely related to mitochondrial autophagy include Parkin, a widely expressed E3 ubiquitin ligase that has been shown in many experiments to regulate the process of mitochondrial autophagy [16]. With the initiation of mitochondrial autophagy, Parkin can be recruited by PINK1 to the outer mitochondrial membrane and interacts with LC3–phospholipid coupling (LC3-II) to promote mitochondrial elimination [17]. As autophagy is a crucial link in ensuring mitochondrial quality, it is often an important aspect of pathogenesis in many diseases when the body undergoes dynamic changes and is subjected to external stimuli.

Mitochondria are dynamic organelles whose normal energy production is closely related to the maintenance of their normal morphology. Fission and fusion, as important components of mitochondrial dynamics, directly affect changes in mitochondrial morphology. Since mitochondria can form a long tubular network, fission can split the tubular mitochondrial network into small cellular fragments, which facilitates the removal of damaged mitochondria through cellular autophagy. In this process, Drp1, a cytoplasmic member of the GTPase dynamin family, plays an important role [18]. When the mitochondrial membrane potential decreases, Drp1 is recruited from the cytoplasm to the outer membrane of mitochondria and binds to different receptors such as mitochondrial fission factor (MFF) and mitochondrial fission 1 (FIS1), thereby mediating mitochondrial rupture [4]. Meanwhile, the post-transcriptional phosphorylation of Drp1 has been shown to be a key substance in the regulation of fission, and the upstream pathways regulating Drp1 have also been demonstrated experimentally [19]. The fusion process involves the mutual fusion of the outer and inner membranes of two mitochondria. It is mainly mediated by the outer membrane fusion proteins mitochondrial fusion protein 1 (Mfn1) and mitochondrial protein 2 (Mfn2) and the inner membrane fusion protein Opa1, allowing for material exchange [20]. When the mitochondrial oxidative phosphorylation (OXPHOS) process can produce active ATP in a normal way, the fusion process will be enhanced, and its specific regulatory mechanism requires further research. In conclusion, normal mitochondrial dynamics can only be maintained when the fusion and cleavage processes are in equilibrium, thus ensuring a normal energy supply.

**Figure 1:**
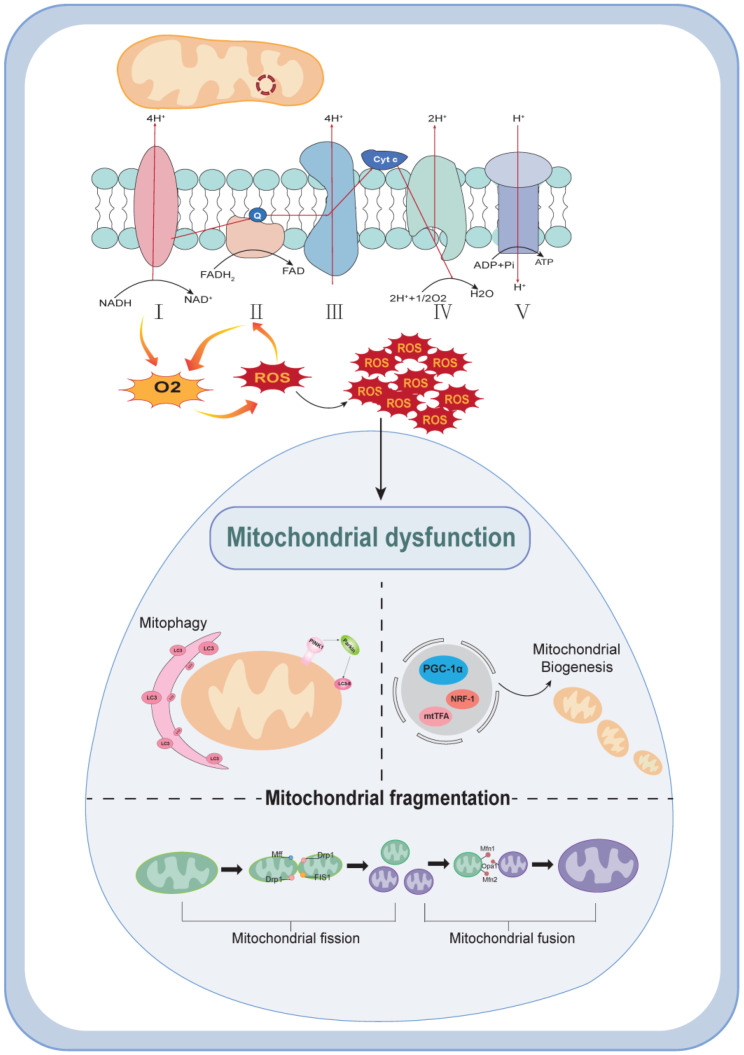
The mechanism of mitochondrial dysfunction. Reactive oxygen species (ROS) are mainly generated by the mitochondrial electron respiration chain. When a large amount of reactive oxygen species is produced, cells maintain dynamic balance by regulating mitochondrial biogenesis and autophagy. In terms of dynamics, mitochondria maintain their normal morphology through Drp1-mediated cleavage and Mfn1-, Mfn2-, and Opa1-mediated fusion. The disruption of any of the above links can lead to mitochondrial dysfunction.

These four aspects work together to maintain mitochondrial homeostasis while affecting ATP and ROS production, and when their homeostasis is disrupted, it leads to corresponding pathological changes in the organism (Figure 1).

## 3. Progression and Mechanisms of Diabetic Nephropathy

As a chronic progressive disease, the development process of DKD can be understood from two points of view: changes in renal structure and function. Structurally, the early stages of DKD are characterized by an increase in kidney volume and partial thickening of the basement membrane. As the disease progresses, the basement membrane will further thicken and show the characteristics of mesangial dilatation. In end-stage renal disease, renal fibrosis is the main manifestation. Functionally, in the early stages of the disease, small arteries are transiently dilated due to hyperglycemia, resulting in an increase in the glomerular filtration rate (eGFR). As the disease progresses, the glomeruli and tubules are further damaged by the constant stimulation of inflammation and toxic metabolites, resulting in the persistence of albuminuria and a progressive decrease in eGFR [2]. However, not all DKDs develop one by one according to this process, and the glomerular filtration rate and albuminuria production should not be used as the sole criteria for judging the progression of DKD [21].

**Figure 2:**
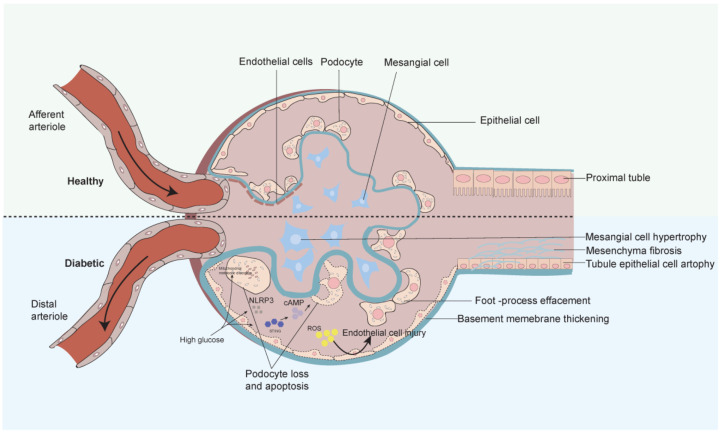
The progression of diabetic nephropathy. Under normal circumstances, the glomerular filtration membrane is composed of an inner to outer layer of endothelial cells, basement membrane, and podocytes. A high-glucose environment can activate the cGAS–STING pathway and NLRP3 inflammasome, leading to pathological changes such as podocyte loss and apoptosis, epithelial cell damage, and foot process fusion. There will be manifestations of tubular cell proliferation, hypertrophy, and interstitial fibrosis in the renal tubules. These are all related to the production of proteinuria.

Since the pathogenesis of DKD is diverse, in this review, we will look at both glomerulosclerosis and tubular injury (Figure 2). The glomerular filtration membrane consists of three layers of structures, namely, from the inside out, the endothelial cell layer, the basement membrane layer, and the epithelial cell layer. Epithelial cells, also known as podocytes, are terminally differentiated cells in the glomerulus, connected by the cell body and pods through the cytoskeleton. They play an important role in maintaining normal filtration barriers and are also important factors in the pathogenesis of DKD [22]. The abundance of mitochondria in the glomerulus provides energy for its proper functioning and maintains the peduncle structure through energy-dependent motorized protein filaments in the peduncle [23]. When metabolic pathways in the body are disturbed, mitochondrial homeostasis breaks down and pathological changes such as podocyte loss occur [24]. Glomerular basement membrane thickening, a characteristic manifestation of early DKD, is caused by the early high-glucose activation of podocytes, causing hemodynamic changes that lead to an increase in the extracellular matrix. At present, DKD is also considered a chronic inflammatory disease. In the early stage of DKD, it was found that high sugar can activate the cGAS-STING pathway composed of interferon gene-stimulating factor (STING) and cyclic GMP AMP (cGAMP) synthase (cGAS), leading to foot process fusion and basement membrane thickening [25]. At the same time, DKD can also cause the activation of the inflammatory body NLRP3 and lead to the scorch death of the foot cells, which is related to the production of proteinuria [26]. In the later stage of the disease, the fused foot processes will gradually disappear, leading to the depletion of foot cells and glomerulosclerosis. In addition to the above, available evidence suggests that damage to the endothelial cells (GEC) also accelerates DKD progression in the presence of intact podocytes. ROS in ECs are mainly derived from mitochondria, NADPH oxidase (NOX), endothelial NOS (eNOS) uncoupling, and xanthine oxidase (XO) [27]. Due to mitochondrial dysfunction in endothelial cells, ROS lead to endothelial cell damage and apoptosis. Due to the close relationship between the extensive filtration function of GECs and the surface layer of luminal cells (ESL), the significant decrease in ESL in DKD leads to glomerular dysfunction [28]. Moreover, in the glomerular filtration membrane as a whole, abnormal mediator action can cause mutual interference between the cells of the filtration membrane, contributing to the development of DKD [29].

In recent years, the focus of DKD research has been partially shifted towards renal tubular pathology. As an important unit of renal reabsorption, renal tubular epithelial cells (TECs) undergo multiple injuries, leading to irreversible pathological changes in the renal tubules. When patients present with microalbuminuria, the severity of lesions occurring in the tubulointerstitium is found to be rather higher than in the glomeruli [30]. In the early stages of DKD, increased glucose transport to the proximal renal tubules triggers secondary hypertrophy and hyperplasia of the tubules, and a high degree of reabsorption also leads to hypoxia in the tubules [31]. As the terminal pathway of DKD, renal tubulointerstitial fibrosis is closely related to epithelial–mesenchymal transition (EMT), energy balance imbalance, and the activation of inflammasomes. Under high glucose stimulation, TECs increases TGF-β1 secretion to promote mesangial fibrosis and increase extracellular matrix production [32]. Normal oxidative phosphorylation and fatty acid metabolism become important due to the high energy demands of the renal tubules. Mitochondria are important producers of ATP, and their mass damage in DKD leads directly to oxidative stress and apoptosis in TECs [33]. Meanwhile, the ectopic deposition of lipids and fatty acid induction can further aggravate proximal tubular injury, leading to vascular thinning and ultimately glomerular fibrosis [34]. In terms of inflammation, high glucose levels are associated with high blood glucose levels. From an inflammatory perspective, high glucose specifically induces IL-1 in the proximal renal tubules, α increases and forms a signaling cascade reaction with inflammasomes to promote the progression of DKD [35].

## 4. Diabetic Nephropathy and Mitochondrial Dysfunction

### 4.1. DKD and Mitochondrial Biogenesis

Mitochondrial biogenesis is a complex self-protective mechanism that serves as an important pathway for the production of new mitochondria and provides a reserve for energy requirements. The kidney is one of the organs with a high energy demand in the human body, and the enhancement of mitochondrial biogenesis under DKD may have a protective effect. In contrast, mitochondrial biogenesis is simultaneously regulated by a network of multiple factors.

In DKD, podocytes exhibit diffuse peduncle fusion and apoptosis, and further observations reveal morphological changes such as swelling and the loss of cristae in mitochondria [36]. A reduction in PGC-1α, an important factor involved in mitochondrial biogenesis, has been shown to promote DKD. AMPK is a key link in the regulation of cellular metabolism, and it can directly increase the level of PGC-1α expression to promote mitochondrial biogenesis and thus slow down the progression of DKD [37]. The pathway is also known as the histone pathway. In addition to this pathway, the histone deacetylase Sirt1 can activate PGC-1α in an AMPK-independent manner to ameliorate podocyte injury [38]. Subsequently, the activated PGC-1α can trans-activate nuclear respiratory factor 2 (Nrf2) and further upregulate mitochondrial transcription factor A (TFAM) to increase mtDNA content. For example, the upregulation of Nrf2/TFAM by astragaloside (AS-IV) in podocytes increased mitochondrial biogenesis and reduced oxidative stress [39].

Mitochondrial glycerol 3-phosphate dehydrogenase (mGPDH), a link in the mitochondrial respiratory chain, is markedly reduced in DKD mice and correlates with podocytopathic manifestations. Experimental sequencing revealed that mGPDH can regulate RAGE signalling by inhibiting S100A10 to restore mitochondrial biogenesis [24]. Due to the complexity of the regulatory network, many factors and receptors can be involved in this regulation. For example, Sestrin2 activates the AMPK/Sirt1/PGC-1α pathway [40], and the downregulation of the reninogen receptor PRR can improve DKD by normalizing PGC-1α levels [9].

The proximal renal tubules are responsible for reabsorbing most of the nutrients, vitamins, inorganic salts, etc., filtered by the glomeruli. During this process, normal mitochondria guarantee the protection of tubules from damage. In response to high-glucose stimulation, renal tubules undergo a series of pathological changes including epithelial cell regeneration and vesicular degeneration. The previously mentioned PGC-1α remains important in renal tubular mitochondrial biogenesis. It can activate CREB in the PGC1-α gene promoter region after binding to adiponectin (APN) and adiponectin receptor (AdipoR) to promote mitochondrial biogenesis [7]. Based on the importance of mitochondrial ribosomal protein 12 (MRPL12) in mitochondria, research has found that under high-glucose conditions, ubiquitin ligase (CLU3) can ubiquitinate MRPL12, leading to impaired mitochondrial biosynthesis and exacerbating renal tubular injury [41]. Sodium–glucose cotransporter (SGLT) is an important carrier of proximal tubule reabsorption. Inhibiting the expression of SGLT2 can reduce kidney injury and enhance the expression of PGC-1α and Nrf2 [42].

### 4.2. DKD and ROS Generation

ROS production is an important cause of diabetic microangiopathy, and excessive ROS can lead to oxidative stress. Available experiments have shown that DKD increases ROS production, which, in turn, can inversely exacerbate mitochondrial dysfunction, worsening DKD [43].

Due to the non-renewable nature of podocytes, excessive ROS will lead to podocyte apoptosis. ROS production in vivo mainly comes from the mitochondrial electronic respiratory chain. mtDNA, as an important material encoding the subunits of the respiratory chain, lacks a mature self-repair mechanism and is susceptible to interference from the external environment. The anchoring protein AKAP1 located on the outer membrane of mitochondria can reduce mtDNA replication and TFAM in podocytes by recruiting PKC-phosphorylated Larp1, leading to the destruction of complex II in ETC [44]. Also under DKD, a reduction in the mitochondrial DNA repair enzyme OGG1 increases ROS production [43]. In addition to this, podocytes require ATP produced by OXPHOS to maintain normal function, of which fatty acid oxidation (FAO) accounts for a large proportion. In FAO, lipid disorders upregulate CD36 expression to increase the uptake of free fatty acids (FFAs), which can accelerate apoptosis in podocytes [45]. Studies have found that there is a large amount of lipid accumulation in the kidneys of db/db mice. Besides regulating biogenesis, the PGC-1α mentioned above can also be upregulated to improve metabolism and reduce ROS production through AMPK/PGC-1α [46]. The inflammatory body NLRP3 is involved in the release of innate immune IL-1β in the cytoplasm and is associated with DKD. When NLRP3 activation is inhibited, IL-1β/ROS/NF-κB p65, a pathway associated with lipid accumulation in DKD, can be inhibited to reduce lipid accumulation in podocytes [47]. NOX4 belongs to the NADPH oxidase family, and it has been proven that high glucose can enhance its expression and cause excessive ROS production. When ginsenoside Rb1 is administered to reduce NOX4 activity, mitochondrial function and structure are alleviated [48]. There are many other factors affecting ROS production, such as the accumulation of ceramide in foot cells under high glucose [49]. In addition, the downregulation of SETD6 can activate Nrf2-Keap1 to ameliorate oxidative stress [50].

The renal tubule is the site of the transmembrane transport of substances and contains a large number of mitochondria to provide energy for this. In hyperglycemia, ROS production can be increased by generating excess NADH and FADH2 through the tricarboxylic acid cycle. Under the stimulation of excessive ROS, mitochondrial structure destruction and decreased membrane potential can lead to cell apoptosis, accompanied by morphological disorders of renal tubules. An increase in nicotinamide adenine dinucleotide (NAD+), as an important link in repairing mtDNA, can have a protective effect on DKD. α-Klotho is an anti-aging protein primarily expressed in renal tubules that increases NAD+ content by inhibiting enzymes synthesized from NAD+ to reduce proximal tubular injury [51]. The renal tubules prioritize OXPHOS to provide energy, and the downregulated MRPL12 in DKD can bind with Nrf2 upstream to exert a positive regulatory effect on OXPHOS [52]. In recent years, there has been growing interest in the effects of hypoxia on the renal tubule. The generation of energy under hypoxic conditions is replaced by anaerobic oxidation involving lactic acid. At this time, the lactate dehydrogenase LADH in the renal tubules binds to NADH to mediate ROS generation, which may be related to renal fibrosis [53]. In addition, high sugar can cause the non-selective pore mPTP to open and disrupt the mitochondrial membrane potential, which can be blocked by tumor necrosis factor receptor-associated protein 1 (TRAP1) to reduce cell apoptosis [54]. The mPTP opens and disrupts the mitochondrial membrane potential. At the same time, activation of the saline corticosteroid receptor (MR) in DKD can reduce ROS production after its blockade [55]. There are other experiments that have been demonstrated to reduce ROS production in renal tubules, such as activated protein c, which can both reduce ROS and inhibit Nlrp3 activation, but the exact mechanism remains to be investigated [56].

### 4.3. DKD and Mitochondrial Autophagy Disorders

Mitochondrial autophagy is a protective process that includes autophagosome formation, fusion with lysosomes, and the degradation of contents by hydrolytic enzymes. It is crucial for maintaining normal cellular function and intracellular homeostasis. Mitochondrial autophagy may increase in the early stages of DKD pathogenesis [57]. However, as the disease progresses, ROS production and the effects of various pathways lead to mitochondrial autophagy dysfunction.

Regarding podocyte damage and loss—a key factor affecting DKD—cardiolipin (CL) plays an important role in maintaining mitochondrial membrane function. In the DKD environment, CL can be abnormally reshaped by elevated haemolytic phosphatidyltransferase 1 (ALCAT1), leading to excessive ox-CL and inhibition of autophagy and cascading cell death [58]. Due to the importance of mitochondrial morphology for maintaining normal function, giant mitochondria formed by a reduction in autophagy flux were found in an experiment simulating the diabetes environment in vitro, which also caused podocyte damage [59]. PINK1/Parkin is an important pathway of mitochondrial autophagy, and its activation can enhance co-localization with LC3 to protect mesangial cells [60]. In addition, Parkin can ubiquitinate downstream proteins and reduce ROS production in podocytes to reduce apoptosis [61]. In DKD patients, it was found that the increase in the autophagy key protein p62 leads to a decrease in autophagy and an increase in apoptosis. By targeting Bcl-2 with baicalin, apoptosis can be reduced or become a new target [62]. The activation of PI3K/AKT/mTOR, a recognized autophagy inhibitory pathway, decreases cellular autophagy [63]. However, podocytes rarely use mTOR for regulation, so new findings have shown that osmotic ion channels (TRPC6) can damage autophagy by activating calpain, as determined in a new study on the relationship between podocyte cytoskeleton and autophagy [64]. All of the above demonstrates the complexity and uniqueness of the regulation of autophagy by podocytes.

As the enriched site of mitochondria, the lack of mitochondrial autophagy in renal tubules is not only related to the aging of renal tubules, but also accelerates the progression of DKD. Research in this area is still limited, and the main regulatory mechanisms are shown in the following areas. As key components involved in mitochondrial autophagy, PINK1 and Parkin have also been extensively studied in renal tubules. In the mouse model induced by HFD/STZ, renal tubular epithelial cell shedding can be observed. After the administration of Huangkui capsules, it was found that they can act on mitochondrial DNA to activate STING1, thereby upregulating PINK1 expression upstream and increasing autophagy [65]. Tumor necrosis factor α TIPE1, a member of the family, is highly expressed in DKD. Further research has found that it damages PINK1/Parkin-mediated mitochondrial autophagy by accelerating the degradation of the mitochondrial inner membrane protein PHB2, ultimately leading to renal tubular fibrosis [66]. Moreover, mitoQ, an antioxidant targeting mitochondria, can restore mitochondrial autophagy through NRF2-mediated PINK transcription, but the exact mechanism needs to be further investigated [33]. In addition, endoplasmic reticulum stress can also promote the progression of DKD. Under HG conditions, inhibiting epoxide hydrolase (sEH) can improve endoplasmic reticulum stress while promoting autophagy [67]. With the development of high-throughput sequencing, it has been found that long-stranded non-coding RNAs (lncRNAs) may be associated with renal tubular injury, but in vivo experiments are still needed to further explore the mechanism [68].

### 4.4. DKD and Mitochondrial Dynamics Disorders

**Figure 3:**
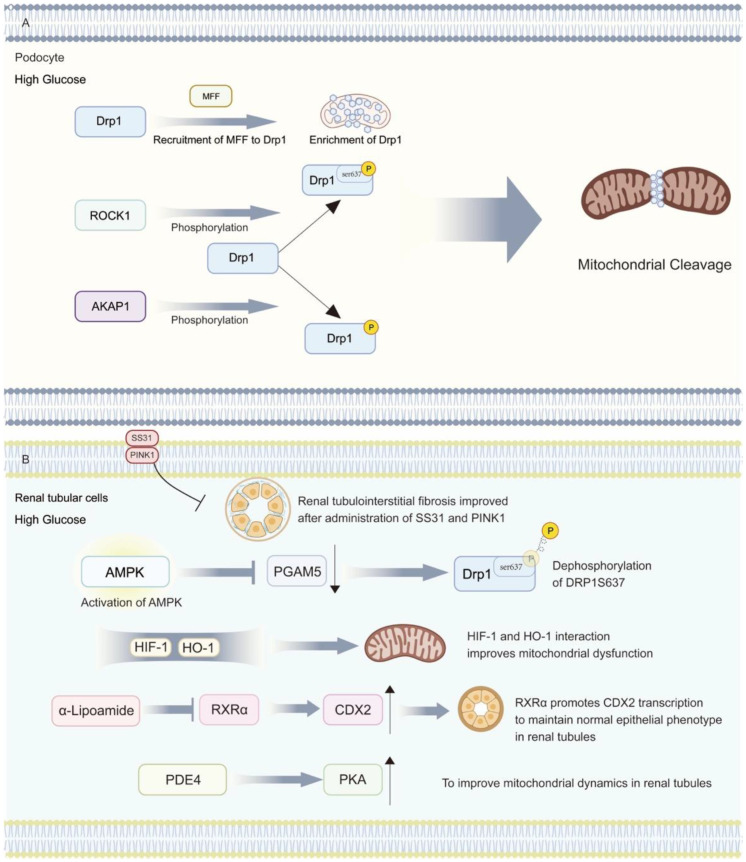
Diabetic kidney disease and mitochondrial dynamics disorder. (**A**) The damage mechanism of mitochondrial lysis in podocytes under a high-glucose environment. (**B**) The protective mechanism of reducing mitochondrial lysis in tubular cells under a high-glucose environment.

Mitochondrial dynamics, including both fusion and fission, are fundamental in adapting to changes in an organism by changing their shape and number. However, in DKD, energetic disturbances lead to an increase in mitochondrial fission and a decrease in fusion, thus contributing to disease progression (Figure 3).

Excessive mitochondrial fission in podocytes is a typical feature of kidney injury. As one of the important proteins involved in mitochondrial fission, Drp1 is recruited by mitochondrial fission factor (MFF) to promote mitochondrial fission. The upregulation of MFF expression and an increase in mitochondrial quantity and length were also observed in DN mice [69]. Drp1 is also subject to a variety of post-translational modifications and can be phosphorylated by elevated AKAP1 in the HG environment, which translocates to mitochondria and promotes cleavage [70]. At the same time, the thromboxane receptor TP can also promote the phosphorylation of Drp1 at the Ser637 site through Rho-associated kinase 1 (ROCK1) in DKD, leading to podocyte injury [71]. And bispecific protein phosphatase (DUSP-1) can inhibit the JNK/MFF pathway during overexpression, reducing the phosphorylation of Mff and thus providing renal protection [19]. From a fusion perspective, Opa1 regulates the fusion of the inner mitochondrial membrane and the morphology of mitochondrial cristae. Opa1 is subject to gene transcription regulated by PGC-1α and can be activated by PKM2 to increase expression so as to maintain mitochondrial stability [72]. In addition, when mitochondria are stressed in DKD, when activated OMA1 hydrolyses Opa1, leading to increasing podocyte damage. This condition can be alleviated by upregulated SS31 [73]. In conclusion, the inhibition of mitochondrial fission and the promotion of its fusion can better protect podocytes from albumin-induced cellular damage in DKD [74].

In the dynamics of the renal tubule, fibrosis of the tubular interstitium and increased matrix deposition occur under HG conditions, and this phenomenon is correspondingly alleviated when SS31 and PINK1 are administered [75,76]. In vitro experiments on HK2 cells have shown that under HG conditions, phosphorylated DRP1S637 decreased and PGAM5 expression increased. When using AMPK activators, the phosphorylation level of AMPK can be increased, followed by the downregulation of PGAM5 levels to protect cells [77]. Meanwhile, hypoxic conditions should not be overlooked in the progression of DKD. HIF-1, a key factor in attenuating hypoxic injury, can improve mitochondrial dysfunction by interacting with heme oxygenase (HO-1). It is of concern that although HIF-1 is elevated in diabetic kidneys, this is only a compensatory response produced to protect the kidneys [78]. Furthermore, mitochondrial homeostasis is regulated by nuclear genes. RXRα, as an intermediate messenger between mitochondria and nucleus, can promote CDX2 transcription to maintain the normal epithelial phenotype of renal tubules, and this effect in alpha lipoamide promotes and reduces mitochondrial lysis [6]. Meanwhile, the inhibition of phosphodiesterase 4 (PDE4) can reverse the reduction in PKA levels under HG, thereby improving mitochondrial dynamics in the renal tubule and providing a new target for studies [79]. We briefly summarized the effects of mitochondrial dysfunction on cells within the kidney in Table 1.

## 5. Therapeutic Options Targeting Mitochondrial Dysfunction in Diabetic Nephropathy

As mentioned earlier, mitochondrial dysfunction is involved in different aspects of DKD pathology. Although the role played by mitochondrial function in DKD is currently not fully understood, targeting mitochondrial dysfunction may be a very promising strategy for treating DKD. Although some of the current studies have not yet been extended to the clinic, a large number of experiments suggest that targeting mitochondrial dysfunction is a reasonable option (Table 2).

In the mitochondrial biogenesis link, AMPK, as an important molecule in the positive regulation of biogenesis, can activate PGC-1α downstream and also regulate Mfn2 to ameliorate oxidative stress. After the administration of BSTL in db/db mice, the phosphorylation of AMPK in podocytes was restored and proteinuria appeared to be correspondingly reduced [37]. Also, in the development of renal tubulointerstitial fibrosis, Schisandrin B reduces the progression of tubular fibrosis by binding to the AMPKSer172 phosphorylation site [86]. Oxidative stress is an important component in the development of DKD, and many targeted therapies have been focused on it. Nrf2 is a recognized stress-response transcription factor. When its pathway, Nrf2/KEAP1/ARE, is activated by ROS, a large number of antioxidant enzymes (NQO1, SOD, HO-1) are upregulated, providing new targets for treatment [87]. For example, astragaloside targets the upregulation of Nrf2 to reduce apoptosis in podocytes [39]. For renal tubules, conventional antioxidants are less effective due to their inability to be absorbed by mitochondria [88]. When using the mitochondrial-targeted antioxidant mitoQ, Nrf2/PINK1 can be regulated to reduce the cell death caused by renal tubular oxidative stress [33]. In addition to oxidative stress, inflammation is inextricably linked to mitochondrial dysfunction. In previous treatments, the drug cagliflozin, targeting sodium glucose transporter 2 (SGLT2), was shown to reduce the release of inflammatory factors and improve patient survival [89], while nicotinamide riboside (NR), a therapeutic agent targeting mitochondria, can reduce cGAS-STING activation in DKD and improve inflammation [90]. Secondly, large lipid droplets and lipid deposition can be observed in diabetic kidneys, which are significantly associated with ROS production and inflammatory response [91]. NLRP3 inflammatory vesicles can be activated in DKD, increasing lipid accumulation in podocytes and overproducing reactive oxygen species via IL-1β, and their inhibition could be a new therapeutic target [49]. Renal tubular epithelial cells often rely on fatty acid oxidation (FAO) for energy. As a downstream target of minR-21, PPARα can activate factors related to lipid metabolism (CPT1a, ADH, ACOX1), which is particularly important for providing energy to the renal tubule [92]. When the lipid-lowering drug atorvastatin is used, min-21-mediated FAO and thus tubular fibrosis can be improved [93].

In terms of mitochondrial autophagy and its kinetics, granular proteinogen (PGRN) has anti-inflammatory and wound repair functions. A significant decrease in PGRN was observed in DKD, and its increase can positively modulate mitochondrial autophagy, thus revealing a new role of PGRN in maintaining podocyte homeostasis [38]. Also, in T2DM mice, increased salt corticosteroid (MR) expression was found in the renal tubules. MR in turn can be activated with aldosterone action, thereby promoting renal inflammation and fibrosis [94]. Based on this, the study first focused on steroid antagonists. However, to prevent the production of hyperkalemia, the experiment shifted to non-steroidal mineralocorticoid antagonists such as finarenone (FIN), which was found to inhibit MR to restore mitochondrial autophagy [55]. Meanwhile, p66shc, a member of the Shc family, mediates oxidative stress and ROS production, which leads to renal unit hypoplasia [95]. Mitochondrial mass damage caused by the rise of p66Shc was observed in STZ in induced-diabetes rats. It was proven that p66Shc can be used as a therapeutic target to reduce the progress of DKD after GAPE was given [96]. In addition, changes in gut microbiota function and composition have been observed in DKD patients. Through the gut–kidney axis, gut microbiota can promote DKD through various aspects, such as metabolites and immune responses [97]. Therefore, treatments targeting the improvement of intestinal flora will also become a major research direction [98]. Nowadays, there is also a lot of evidence showing the important role of organelles in DKD [99]. Among them, mitochondria-associated endoplasmic reticulum (MAM) act as tight junctions between the outer membrane and endoplasmic reticulum of mitochondria, and their integrity is negatively correlated with DKD progression. Under the protection of the connecting protein PACS-2, MAM homeostasis can be maintained and DKD can be improved [100]. However, the favorable effects demonstrated by the above experiments in cellular or animal models need to be further explored to better determine their safety and stability in clinical applications.

## 6. Conclusions and Expectations

Mitochondrial dysfunction in the kidney, a highly energy-demanding organ, may be closely associated with glomerular and tubular damage in DKD. In normal mitochondrial homeostasis, it maintains kinetic stability through fission and fusion, removes abnormal mitochondria with autophagy, and continuously generates new mitochondria through biogenesis. When homeostasis is disrupted, it can lead to basement membrane thickening and peduncle fusion in the glomerulus and, finally, to the development of irreversible cellular damage and glomerulosclerosis. Secondly, tubular atrophy and interstitial fibrosis can occur in the renal tubules. These may manifest clinically as proteinuria and a progressive decline in renal function. However, our current understanding of the role of mitochondria in the progression of DKD remains to be further understood. First, the criteria for assessing mitochondrial dysfunction in cells are not standardized, and different approaches may lead to different determinations [101]. Second, we lack a more detailed understanding of the role that mitochondrial dysfunction plays in each component of DKD. On the therapeutic side, although we have demonstrated in some animal experiments that improving mitochondrial function can delay the progression of DKD, a further evaluation of its therapeutic efficacy and safety is still lacking, so there are no drugs targeting mitochondrial dysfunction in DKD yet. Therefore, we still need more experiments to offer further validation. In conclusion, with proper experimental design and theoretical foundations, the relationship between DKD and mitochondria will be more fully elucidated, and the targeting of mitochondrial dysfunction will be a promising research direction in the treatment of DKD.

## Figures and Tables

**Table 1:** Effect of mitochondrial dysfunction on cells within the kidney.

Kidney Cells	Abnormalities Caused by Mitochondrial Dysfunction	References
Mesenchymal cells	Metabolic disorders Increased oxidative stress Fibrosis	[80]
Glomerular endothelial cells	Inflammatory response Endothelial barrier damage	[81,82]
Podocytes	Impaired filtration function Increased apoptosis Cytoskeletal changes	[47,83,84]
Renal tubular cells	Impaired reabsorption Cell damage and apoptosis Metabolic imbalances	[85]

**Table 2:** Potential therapeutic approaches to target mitochondrial dysfunction in DKD.

		Mechanism	DKD Model
Mitochondrial biogenesis and dynamics	BaoShenTongLuo (BSTL)	BTSL restored phosphorylation of AMPK and reduced podocyte apoptosis, suppressed excessive cellular ROS production, and reversed the decrease in MMP that was observed under HG conditions	db/db mice and mouse podocytes line MPC-5
	Schisandrin B (Sch B)	Reduction of tubular fibrosis progression by binding to the AMPKSer172 phosphorylation site	db/db mice and the human proximal tubular cell line HK-2
	Grape seed proanthocyanidin extract	Target and downregulate p66Shc	Streptozotocin-induced diabetic mice
Mitochondria-targeted antioxidant	Astragaloside IV	Ameliorate mitochondrial dysfunction by up-regulated Nrf2-ARE/TFAM signaling	Mouse podocytes line
	MitoQ	Regulation of the Nrf2/PINK1 signaling pathway	db/db mice and the human proximal tubular cell line HK-2
Inhibitors of mitochondrial inflammation	Nicotinamide riboside (NR)	Reduced cGAS-STING activation in DKD	db/db mice
	Atorvastatin	Downregulation of miR-21 expression activates PPAR-α to enhance FAO to ameliorate tubular fibrosis	Streptozotocin-induced diabetic mice and mouse renal tubular epithelial cells
Promotes mitochondrial autophagy	finerenone(FIN)	Improvement of mitochondrial autophagy via PI3K/Akt/eNOS signaling pathway	Streptozotocin-induced diabetic mice and the human proximal tubular cell line HK-2

Abbreviations: MMP, mitochondrial membrane potential; AMPK, Adenosine 5′-monophosphate (AMP)-activated protein kinase; Nrf2, Nuclear factor erythroid 2-related factor 2; ARE, anti-oxidant response element; TFAM, transcription factor A; PINK1, PTEN induced putative kinase 1; cGAS, cyclicGMP-AMP synthase; STING, stimulator of interferon genes; PPAR-α, peroxisome proliferators-activated receptors-α; FAO, fatty acid oxidation; PI3K, Phosphoinositide 3-kinase; Akt: PKB, protein kinase B.
